# 伴急性髓系白血病样突变的骨髓增生异常肿瘤患者临床特征及预后分析

**DOI:** 10.3760/cma.j.cn121090-20250513-00228

**Published:** 2025-11

**Authors:** 泽飞 包, 琳琳 刘, 冰 李, 铁军 秦, 泽锋 徐, 士强 曲, 丽娟 潘, 清妍 高, 蒙 焦, 玉娇 贾, 承文 李, 琦 孙, 慧君 王, 志坚 肖

**Affiliations:** 1 中国医学科学院血液病医院（中国医学科学院血液学研究所），血液与健康全国重点实验室，国家血液系统疾病临床医学研究中心，细胞生态海河实验室，天津 300020 State Key Laboratory of Experimental Hematology, National Clinical Research Center for Blood Diseases, Haihe Laboratory of Cell Ecosystem, Institute of Hematology & Blood Diseases Hospital, Chinese Academy of Medical Sciences & Peking Union Medical College, Tianjin 300020, China; 2 天津医学健康研究院，天津 301600 Tianjin Institutes of Health Science, Tianjin 301600, China

**Keywords:** 骨髓增生异常肿瘤, 急性髓系白血病, 基因突变, 预后, 白血病转化, Myelodysplastic neoplasm/Myelodysplastic syndrome, Acute myeloid leukemia, Mutation, Prognosis, Leukemic transformation

## Abstract

**目的:**

分析伴急性髓系白血病（AML）样突变的骨髓增生异常肿瘤（MDS）患者的临床和实验室特征及预后。

**方法:**

收集2016年8月至2024年6月于中国医学科学院血液病医院确诊的1 464例初治成人原发性MDS患者病例资料，回顾性分析伴AML样突变MDS患者临床、分子学特征以及预后情况。

**结果:**

共64例（4.4％）患者伴有AML样突变。与无AML样突变患者相比，伴AML样突变患者年龄更小［50（39, 60）岁对56（45, 65）岁，*P*＝0.001］，女性比例更高（51.6％对35.4％，*P*＝0.009），骨髓原始细胞比例更高［6.5％（3.0％, 10.5％）对2.5％（1.0％, 7.0％），*P*<0.001］，正常核型比例更高（75.0％对48.1％，*P*<0.001），外周血HGB水平更低［73（67, 82）g/L对80（66, 98）g/L，*P*＝0.006］。伴AML样突变组和无AML样突变组中位基因突变数目分别为3（2，4）、2（1，3）个，差异有统计学意义（*P*<0.001）。伴AML样突变组NPM1、DNMT3A、WT1、PTPN11、NRAS、BCOR、FLT3、CEBPA、MYC基因突变率显著高于无AML样突变组（均*P*<0.05），而U2AF1、ASXL1和TP53基因突变率较低（均*P*<0.05）。伴AML样突变患者总生存（OS）期与无AML样突变患者相比差异无统计学意义（*P*＝0.730），中位无白血病生存（LFS）期显著缩短［19（95％*CI*：13～25）个月对46（95％*CI*：38～54）个月，*P*＝0.012］，AML转化率显著增高［2年：（41.7±9.1）％对（10.4±1.1）％，*P*<0.001］。按原始细胞比例分组后，低原始细胞（LB）和原始细胞增多（IB）患者OS、LFS及AML转化率差异均无统计学意义。多因素Cox分析显示，年龄≥60岁和PTPN11突变是影响伴AML样突变患者OS的独立危险因素；DNMT3A突变、PTPN11突变、FLT3突变是伴AML样突变患者发生AML转化的独立危险因素。

**结论:**

伴AML样突变的MDS患者具有独特的临床和分子学特征，并表现出更高的AML转化风险。

## Abstract (en)

**Objective:**

To investigate the clinical, laboratory, and prognostic features of myelodysplastic neoplasm（MDS）patients harboring acute myeloid leukemia（AML）-like mutations.

**Methods:**

We retrospectively analyzed clinical, molecular, and outcome data from 1 464 adults with primary MDS diagnosed at the Institute of Hematology and Blood Diseases Hospital from August 2016 to June 2024.

**Results:**

AML-like mutations were detected in 64 patients（4.4％）. Compared with patients without AML-like mutations, those with AML-like mutations were younger［median 50（*IQR* 39–60）*vs* 56（45, 65）years; *P*＝0.001］, more often female（51.6％ *vs* 35.4％; *P*＝0.009）, had higher bone marrow blast percentage［6.5％（3.0％, 10.5％）*vs* 2.5％（1.0％, 7.0％）; *P*<0.001］, a higher rate of normal karyotype（75.0％ *vs* 48.1％; *P*<0.001）, and lower hemoglobin levels［73（67, 82）g/L *vs* 80（66, 98）g/L; *P*＝0.006］. The AML-like group had a higher number of gene mutations than the non-AML-like group［3（*IQR* 2–4）*vs* 2（1, 3）; *P*<0.001). It was enriched for mutations in NPM1, DNMT3A, WT1, PTPN11, NRAS, BCOR, FLT3, CEBPA, and MYC（all *P*<0.05）and had lower rates of U2AF1, ASXL1, and TP53 mutations（all *P*<0.05）. Overall survival（OS）did not differ between groups（*P*＝0.730）; however, the AML-like group had significantly shorter leukemia-free survival（LFS）［19 months（95％ *CI*: 13–25）*vs* 46 months（95％ *CI*: 38–54）; *P*＝0.012］and a higher 2-year cumulative incidence of AML transformation［（41.7±9.1）％ *vs*（10.4±1.1）％; *P*<0.001］. Within the AML-like group, OS, LFS, and cumulative incidence of AML transformation did not differ between patients with low blasts and those with excess blasts（IB）. Multivariable Cox regression identified age ≥60 years and PTPN11 mutations as independent adverse prognostic factors for OS, while DNMT3A, PTPN11, and FLT3 mutations independently predicted leukemic transformation.

**Conclusion:**

MDS patients harboring AML-like mutations exhibit distinct clinical and molecular features and a higher risk of progression to AML.

骨髓增生异常肿瘤（Myelodysplastic neoplasms, MDS）是一组以外周血血细胞减少、骨髓发育异常及高风险向急性髓系白血病（Acute myeloid leukemia, AML）转化（转白）为特征的异质性髓系肿瘤[1]–[2]。目前，MDS分类体系主要依赖于骨髓细胞形态学特征，未能充分解释MDS临床异质性。建立基于遗传学的分型方法对于理解MDS的发病机制和实现精准化诊疗尤为重要。在最近的一项研究中，Bernard等[3]回顾性分析了3 233例MDS及相关肿瘤患者的基因组特征，并以此把MDS分为18个分子亚型。其中，AML样亚型定义为携带NPM1突变，inv（3）或t（3;3）染色体异常，或者至少检出WT1、FLT3、KMT2A-PTD和MYC突变中的两种。目前尚无研究系统分析AML样亚型MDS患者分子学及临床特征。本研究回顾性分析了我中心伴有AML样突变的原发性MDS患者的临床资料，现报道如下。

## 病例与方法

1. 病例资料：本研究为回顾性队列研究。纳入2016年8月至2024年6月在中国医学科学院血液病医院MDS和MPN诊疗中心确诊并具有完整病例资料的1 464例初治成人原发性MDS患者，所有患者均按WHO（2016）标准[4]诊断。64例患者伴有AML样突变，其中52例携带NPM1突变，7例伴inv（3）或t（3;3）染色体异常，5例至少携带WT1、FLT3、KMT2A-PTD和MYC突变中的两种。

2. 染色体核型分析：短期培养法常规制备染色体标本，应用R显带法进行核型分析。核型描述依据人类细胞遗传学国际命名体制（ISCN2016），并按照修订的国际预后积分系统（IPSS-R）[5]进行染色体核型预后分组。

3. 基因突变二代测序（Next-generation sequencing, NGS）：收集患者骨髓分离单个核细胞提取DNA，使用PCR引物扩增目的基因组，富集后应用Ion Torrent测序平台进行测序。测序后利用CCDS、dbSNP（v138）、HG19、1000genomes、COSMIC等数据库进行生物信息学分析，筛选致病性基因突变位点。具体方法参见本课题组此前已发表文献[6]–[8]。

4. 突变时序分析：基于等位基因突变频率（Variant allele frequency, VAF）对患者进行突变时序分析。患者所有基因突变中VAF值最高且≥5％的突变定义为主克隆突变，与主克隆VAF值相差≥5％的突变为亚克隆突变。当多个基因突变的VAF值差异<5％时，均判定为主克隆突变[6],[9]。

5. 随访和相关定义：随访截止日期为2024年10月25日，随访资料来源于住院病历、门诊病历以及电话随访记录。中位随访时间为26（*IQR*：10，53）个月，其中伴AML样突变患者中位随访时间为15（*IQR*：7，34）个月，无AML样突变患者中位随访时间为26（*IQR*：10，56）个月。失访患者共70例（4.7％），共1 326例（90.6％）患者可随访到治疗方案。总生存（Overall survival, OS）时间定义为自确诊日期至死亡、造血干细胞移植或末次随访日期。无白血病生存（Leukemia-free survival, LFS）时间定义为自确诊日期至死亡、转白、造血干细胞移植或末次随访日期。参照WHO（2016）标准，骨髓原始细胞<5％且外周血原始细胞<2％的患者定义为低原始细胞（LB），骨髓原始细胞≥ 5％或外周血原始细胞≥2％或存在Auer小体则定义为原始细胞增多（IB）。

6．统计学处理：非正态分布的连续变量以中位数和四分位数间距［*M*（*IQR*）］表示，采用Mann-Whitney *U*检验进行组间比较；分类变量以频数和百分比（％）表示，采用卡方检验或Fisher确切概率法进行比较。采用Kaplan-Meier法绘制生存曲线，Cox回归模型进行预后单因素及多因素分析。双侧检验*P*<0.05为差异有统计学意义。应用SPSS 27.0进行统计分析，应用Graphpad Prism 8.0及R 4.4.1绘图。

## 结果

一、伴AML样突变患者的临床特征

1 464例MDS患者中，伴AML样突变患者共64例（4.4％）。如表1所示，与无AML样突变组相比，伴AML样突变组年龄更小（*P*＝0.001），女性比例更高（*P*＝0.009），骨髓原始细胞比例更高（*P*<0.001），外周血HGB水平更低（*P*＝0.006）。两组患者WHO 2016诊断分型（*P*<0.001）、IPSS-R预后分组（*P*＝0.041）和分子学国际预后积分系统（IPSS-M）预后分组（*P*<0.001）各组别分布差异均有统计学意义，而WBC、ANC、PLT等差异无统计学意义。

**表1:** 伴和不伴AML样突变MDS患者的临床及实验室特征比较

临床特征	伴AML样突变组（64例）	无AML样突变组（1 400例）	*P*值
性别［例（％）］			0.009
男性	31（48.4）	904（64.6）	
女性	33（51.6）	496（35.4）	
年龄［岁，*M*（*IQR*）］	50（39, 60）	56（45, 65）	0.001
外周血细胞计数			
WBC［×10^9^/L，*M*（*IQR*）］	2.65（1.92, 5.20）	2.61（1.82, 3.73）	0.346
ANC［×10^9^/L，*M*（*IQR*）］	1.08（0.57, 2.42）	1.10（0.63, 2.01）	0.682
HGB［g/L，*M*（*IQR*）］	73（67, 82）	80（66, 98）	0.006
PLT［×10^9^/L，*M*（*IQR*）］	70（36, 133）	64（33, 130）	0.342
骨髓原始细胞比例［％，*M*（*IQR*）］	6.5（3.0, 10.5）	2.5（1.0, 7.0）	<0.001
WHO 2016诊断分型［例（％）］			<0.001
MDS-SLD	1（1.6）	125（8.9）	
MDS-RS-SLD	0（0）	52（3.7）	
MDS-MLD	14（21.9）	503（36.0）	
MDS-RS-MLD	0（0）	85（6.1）	
MDS-EB1	10（15.6）	291（20.8）	
MDS-EB2	38（59.4）	271（19.4）	
MDS-del（5q）	0（0）	23（1.6）	
MDS-U	1（1.6）	50（3.6）	
IPSS-R预后分组［例（％）］			0.041
极低危	0（0）	58（4.5）	
低危	7（12.5）	344（26.8）	
中危	21（37.5）	384（29.9）	
高危	14（25.0）	280（21.8）	
极高危	14（25.0）	219（17.0）	
IPSS-M预后分组［例（％）］			<0.001
极低危	0（0）	35（2.7）	
低危	1（1.8）	248（19.4）	
中低危	0（0）	204（15.9）	
中高危	5（9.1）	197（15.4）	
高危	25（45.5）	295（23.0）	
极高危	24（43.6）	301（23.5）	
治疗方案［例（％）］^a^			<0.001
等待与观察	0（0）	69（5.4）	
促造血±输血治疗	3（5.3）	165（13.0）	
免疫调节/抑制剂±促造血治疗	2（3.5）	383（30.2）	
去甲基化治疗	12（21.1）	271（21.4）	
联合化疗	8（14.0）	48（3.8）	
造血干细胞移植	30（52.6）	251（19.8）	
中药	0（0）	63（5.0）	
临床试验	2（3.5）	19（1.5）	

**注** MDS：骨髓增生异常肿瘤；AML：急性髓系白血病；MDS-SLD：MDS伴单系血细胞发育异常；MDS-RS-SLD：MDS伴环状铁粒幼红细胞伴单系血细胞发育异常；MDS-MLD：MDS伴多系血细胞发育异常；MDS-RS-MLD：MDS伴环状铁粒幼红细胞伴多系血细胞发育异常；MDS-EB1：MDS伴原始细胞增多1型；MDS-EB2：MDS伴原始细胞增多2型；MDS-del（5q）：MDS伴单纯del（5q）；MDS-U：MDS不能分类；IPSS-R：修订的国际预后积分系统；IPSS-M：分子学国际预后积分系统。^a^1 326例患者可随访到治疗方案，其中伴AML样突变组57例，无AML样突变组1 269例

二、伴AML样突变患者的遗传学特征

1. 细胞遗传学特征：伴AML样突变组中56例（87.5％）患者有可评估的染色体核型，其正常核型比例显著高于无AML样突变组（75.0％对48.1％，*P*<0.001）。异常核型中inv（3）最常见（5例，8.9％），其次是+8（3例，5.4％）和t（3;3）（2例，3.6％）。伴AML样突变组复杂核型检出率显著低于无AML样突变组（0对16.0％，*P*＝0.001）。

2. 分子遗传学特征：伴AML样突变组中位基因突变数目为3（2, 4）个，无AML样突变组为2（1, 3）个，差异有统计学意义（*P*<0.001）。伴AML样突变组中发生频率较高（突变检出率≥5％）的基因突变依次是：NPM1（52例，81.3％）、DNMT3A（16例，25.0％）、WT1（15例，23.4％）、PTPN11（13例，20.3％），NRAS（12例，18.8％），BCOR（11例，17.2％）、FLT3（11例，17.2％）和SF3B1（5例，7.8％）（图1）。其中NPM1（69.2％）、DNMT3A（81.3％）、BCOR（72.7％）、SF3B1（80.0％）突变多为主克隆突变，而WT1（66.7％）、PTPN11（84.6％）、NRAS（91.7％）、FLT3（100.0％）突变多为亚克隆突变。除NPM1突变外，伴AML样突变组DNMT3A（25.0％对7.9％，*P*<0.001）、WT1（23.4％对0.9％，*P*<0.001）、PTPN11（20.3％对1.4％，*P*<0.001）、NRAS（18.8％对4.0％，*P*<0.001）、BCOR（17.2％对4.8％，*P*<0.001）、FLT3（17.2％对0.8％，*P*<0.001）、CEBPA（4.7％对1.0％，*P*＝0.035）、MYC（4.7％对0.1％，*P*<0.001）基因突变率也显著高于无AML样突变组，而U2AF1（1.6％对23.0％，*P*<0.001）、ASXL1（3.1％对21.3％，*P*<0.001）和TP53（1.6％对9.5％，*P*＝0.031）基因突变率较低（图2）。伴AML样突变患者中，IB组患者基因突变数目较LB组患者更多［3（2, 4）个对2（1, 3）个，*P*＝0.005］，但各基因突变检出率两组间差异均无统计学意义。

**图1:**
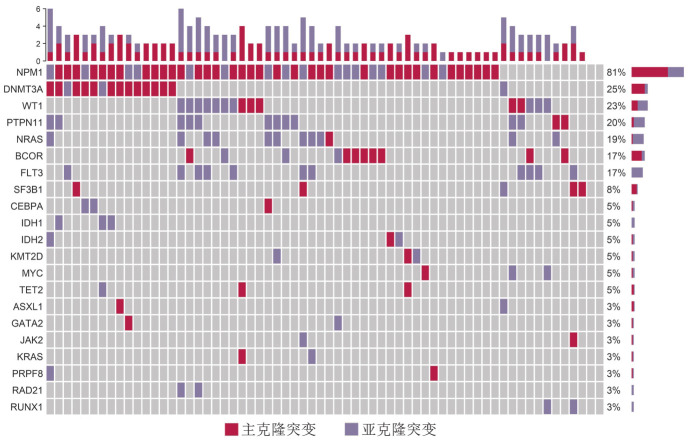
64例伴AML样突变MDS患者基因突变谱 **注** MDS：骨髓增生异常肿瘤；AML：急性髓系白血病

**图2:**
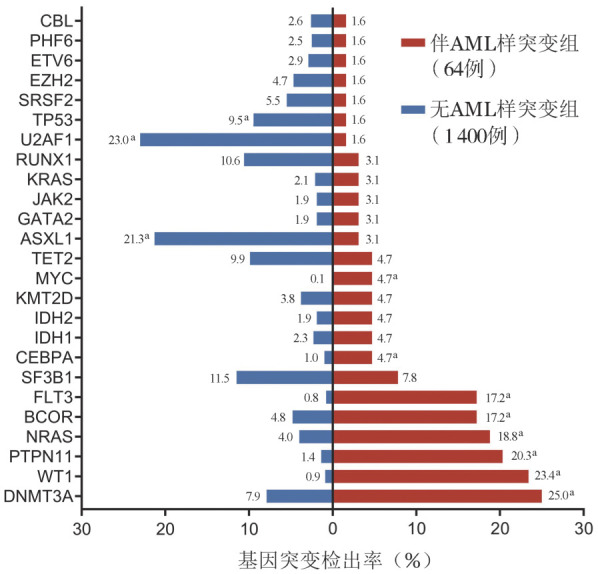
伴和不伴AML样突变MDS患者基因突变检出率比较（不包括NPM1）（^a^*P*<0.05） **注** MDS：骨髓增生异常肿瘤；AML：急性髓系白血病

在转白的患者中，6例伴AML样突变患者有连续的骨髓穿刺及NGS结果（图3）。6例患者在转白时NPM1基因突变VAF均较确诊时增高，4例患者获得至少1个新的基因突变，包括WT1、FLT3、IDH1、NRAS等基因突变。6例患者病程中均未出现新的染色体核型异常。因具有连续样本的患者例数较少未行进一步统计学分析。

**图3:**
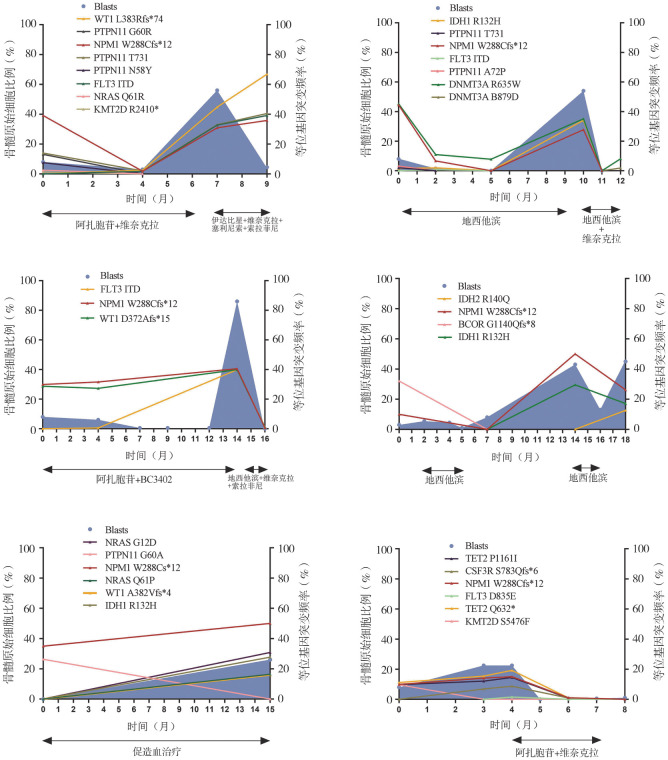
6例发生白血病转化的伴AML样突变MDS患者骨髓原始细胞比例和突变负荷的动态演变过程 **注** 蓝色阴影代表骨髓原始细胞（Blasts）百分比，折线图代表各基因突变等位基因突变频率随时间变化趋势。MDS：骨髓增生异常肿瘤；AML：急性髓系白血病

三、伴AML样突变患者的预后分析

伴AML样突变患者中位OS时间为48（95％*CI*：5～91）个月，无AML样突变患者中位OS时间为48（95％*CI*：39～57）个月，两组间差异无统计学意义（*P*＝0.730）（图4A）。伴AML样突变患者中位LFS时间为19（95％*CI*：13～25）个月，显著短于无AML样突变组的46（95％*CI*：38～54）个月（*P*＝0.012）（图4B）。伴AML样突变组累积转白率显著增高（*P*<0.001），其1年、2年转白率分别为（27.6±6.5）％、（41.7±9.1）％，而无AML样突变组1年、2年转白率分别为（6.5±0.8）％、（10.4±1.1）％（图4C）。伴AML样突变患者中，LB组与IB组OS（*P*＝0.970）、LFS（*P*＝0.580）及累积转白率（*P*＝0.151）差异均无统计学意义（图4D～F）。

**图4:**
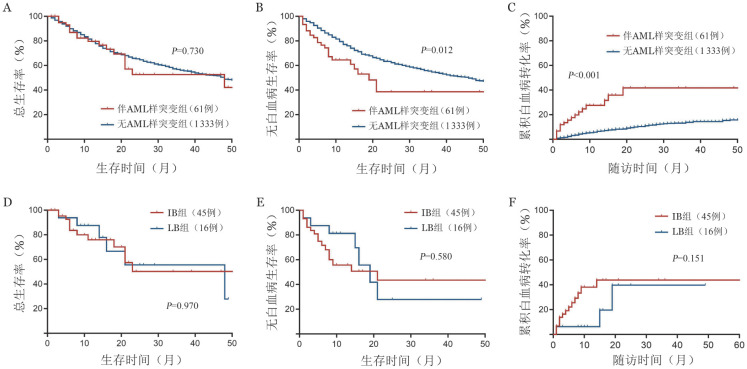
伴AML样突变MDS患者预后分析 **A** 伴AML样突变组与无AML样突变组总生存曲线比较；**B** 伴AML样突变组与无AML样突变组无白血病生存曲线比较；**C** 伴AML样突变组与无AML样突变组累积白血病转化率比较；**D** 伴AML样突变患者中LB与IB亚组总生存曲线比较；**E** 伴AML样突变患者中LB与IB亚组无白血病生存曲线比较；**F** 伴AML样突变患者中LB与IB亚组累积白血病转化率比较 **注** MDS：骨髓增生异常肿瘤；AML：急性髓系白血病；LB：低原始细胞；IB：原始细胞增多

四、伴AML样突变患者的预后影响因素分析

在伴AML样突变患者中，将年龄、性别、IPSS-R预后分组及突变检出率≥5％的基因突变分别行Cox单因素分析，将单因素分析中*P*<0.1的因素纳入Cox多因素分析。结果显示年龄≥60岁（*HR*＝2.679, 95％*CI*：1.020～7.033，*P*＝0.045）和PTPN11突变（*HR*＝3.079，95％*CI*：1.123～8.447，*P*＝0.029）是影响伴AML样突变患者OS的独立危险因素（表2）。

**表2:** 影响伴AML样突变MDS患者总生存的单因素和多因素分析

变量	单因素分析		多因素分析	
	*HR*（95％*CI*）	*P*值	*HR*（95％*CI*）	*P*值
年龄≥60岁	2.840（1.119～7.212）	0.028	2.679（1.020～7.033）	0.045
男性	1.036（0.406～2.645）	0.940		
IPSS-R预后分组				
极低危/低危/中危	参照			
高危/极高危	1.015（0.326～3.165）	0.979		
NPM1突变	1.104（0.319～3.822）	0.876		
DNMT3A突变	2.773（1.045～7.359）	0.041	2.425（0.888～6.625）	0.084
WT1突变	0.991（0.284～3.462）	0.989		
PTPN11突变	2.846（1.061～7.633）	0.038	3.079（1.123～8.447）	0.029
NRAS突变	1.783（0.635～5.009）	0.272		
BCOR突变	0.436（0.057～3.321）	0.423		
FLT3突变	2.248（0.708～7.142）	0.169		
SF3B1突变	2.038（0.581～7.148）	0.266		

**注** MDS：骨髓增生异常肿瘤；AML：急性髓系白血病；IPSS-R：修订的国际预后积分系统

我们进一步探讨了各因素对转白的影响，Cox多因素分析结果显示DNMT3A突变（*HR*＝3.225，95％*CI*：1.139～9.126，*P*＝0.027）、PTPN11突变（*HR*＝3.237，95％*CI*：1.148～9.121，*P*＝0.026）、FLT3突变（*HR*＝3.261，95％*CI*：1.045～10.175，*P*＝0.042）是伴AML样突变患者转白的独立危险因素（表3）。

**表3:** 影响伴AML样突变MDS患者白血病转化的单因素和多因素分析

变量	单因素分析		多因素分析	
	*HR*（95％*CI*）	*P*值	*HR*（95％*CI*）	*P*值
年龄≥60岁	1.021（0.358～2.914）	0.969		
男性	0.801（0.306～2.094）	0.651		
IPSS-R预后分组				
极低危/低危/中危	参照			
高危/极高危	0.830（0.286～2.405）	0.731		
NPM1突变	0.697（0.198～2.450）	0.574		
DNMT3A突变	2.579（0.976～6.813）	0.056	3.225（1.139～9.126）	0.027
WT1突变	1.061（0.302～3.726）	0.926		
PTPN11突变	3.503（1.255～9.778）	0.017	3.237（1.148～9.121）	0.026
NRAS突变	1.420（0.462～4.364）	0.540		
BCOR突变	0.275（0.036～2.080）	0.211		
FLT3突变	2.486（0.866～7.132）	0.090	3.261（1.045～10.175）	0.042
SF3B1突变	0.756（0.100～5.719）	0.786		

**注** MDS：骨髓增生异常肿瘤；AML：急性髓系白血病；IPSS-R：修订的国际预后积分系统

## 讨论

本研究我们发现，相较于无AML样突变患者，伴AML样突变MDS患者确诊时相对年轻，女性比例更高，骨髓原始细胞比例更高，贫血更严重，与Bernard等[3]结果基本一致。在细胞遗传学方面，伴AML样突变组正常核型比例更高，未检出复杂核型。在分子遗传学异常方面，伴AML样突变组NPM1、DNMT3A、WT1、PTPN11、NRAS、BCOR、FLT3、CEBPA、MYC基因突变率显著高于无AML样突变组，而U2AF1、ASXL1和TP53基因突变少见。

NPM1突变是该亚组中最常见的基因突变。NPM1突变见于约三分之一的成人AML患者中[10]，但在MDS中少见[11]。多项研究表明，伴NPM1突变的MDS患者表现出类似于NPM1突变AML的临床特征，包括发病相对年轻、女性患者较多、通常为正常核型、CD34阴性等，并且倾向于迅速进展为AML[12]–[15]。本研究利用连续NGS检测验证了NPM1突变在MDS患者转白过程中的重要作用。信号转导相关基因突变（如NRAS、PTPN11、FLT3等）通常发生在MDS晚期，并与疾病进展和不良预后相关[16]–[20]。我们发现这类突变在伴AML样突变MDS患者中具有较高的检出率，且多为亚克隆突变。连续NGS结果也提示，部分患者在转白时出现新的信号转导相关突变。因此，当患者伴发此类突变时需警惕疾病进展。

本研究显示，伴AML样突变组转白风险明显升高，2年转白率为（41.7±9.1）％，与Bernard等[3]的研究结果基本一致。但Bernard等[3]的研究发现，伴AML样突变组OS时间显著缩短，中位OS时间仅有0.9年。而我们的结果显示，伴AML样突变组中位OS时间为48个月，与无AML样突变组患者相比差异无统计学意义。我们推测导致这一差异的可能原因包括：①本研究对接受异基因造血干细胞移植的患者进行了删失处理，其中伴AML样突变组中52.6％的患者因移植删失，与Bernard等[3]对OS的定义存在差异；②不同队列中患者基因组特征存在差异，如本课题组此前的研究发现[21]，我中心MDS患者NPM1突变频率明显高于西方国家患者。

对伴AML样突变患者行多因素分析发现，年龄≥60岁和PTPN11突变是伴AML样突变患者的独立预后不良因素，而DNMT3A突变、PTPN11突变、FLT3突变为伴AML样突变患者转白的独立危险因素。PTPN11属于RAS通路相关基因，在MDS中罕见。Makishima等[19]发现相较于高危MDS，FLT3、PTPN11、WT1、IDH1、NPM1、IDH2和NRAS这7个基因突变在继发性AML中高度富集，被认为是高风险向AML进展的标志。黄慧君等[20]发现，PTPN11突变在MDS中为独立预后不良因素。本研究结果显示，PTPN11突变在伴AML样突变患者中富集，并与较短的OS及高风险转白相关。以上结果提示，特定的共突变模式与伴AML样突变患者疾病进展和预后相关，有待大样本量研究进一步分析。

此外，我们进一步探讨了原始细胞比例在伴AML样突变患者中的意义。本研究显示，按原始细胞比例分组后，LB和IB组患者OS、LFS及转白率差异均无统计学意义，与Bernard等[3]的研究结果基本一致。近年来，许多研究表明特定遗传学异常的预后价值可能超过了原始细胞比例[22]–[23]。因此，第5版WHO分类[1]对于重现性遗传学异常定义的AML（除外AML伴BCR::ABL1融合基因和AML伴CEBPA突变）取消了原始细胞阈值的限定。2022年国际共识分类（International Consensus Classification, ICC）标准[2]则提出原始细胞比例≥10％作为诊断AML伴重现性遗传学异常（除外AML伴BCR::ABL1融合基因）的临界值，并将原始细胞比例在10％～19％的患者定义为MDS/AML，可根据具体情况选择采取MDS或AML的治疗方案。Montalban-Bravo等[13]和Gener-Ricos等[14]的研究均显示，接受AML样化疗的NPM1突变MDS患者完全缓解率和OS均显著优于接受去甲基化治疗的患者。未来随着MDS基因层面研究的不断深入，基于遗传学的分型方法将有助于识别这类可能从更积极的治疗方案中获益的患者，进一步驱动个体化、精准化治疗进展。

综上，本研究结果证实伴AML样突变的MDS患者具有独特的临床和分子学特征，并表现出更高的转白风险。特定的共突变模式与疾病进展和预后显著相关，而原始细胞分组未能对患者结局进行分层。本研究存在以下不足：①由于在随访中无法获得所有患者的后续治疗方式，未能就治疗方案的影响进行分析；②因具有连续标本的患者例数过少未对克隆演化进行统计学分析；③作为单中心、回顾性研究，不可避免存在偏倚。本研究结论仍需多中心研究加以证实。

## References

[1] Khoury JD, Solary E, Abla O (2022). The 5th edition of the World Health Organization Classification of Haematolymphoid Tumours: Myeloid and Histiocytic/Dendritic Neoplasms[J]. Leukemia.

[2] Arber DA, Orazi A, Hasserjian RP (2022). International Consensus Classification of Myeloid Neoplasms and Acute Leukemias: integrating morphologic, clinical, and genomic data[J]. Blood.

[3] Bernard E, Hasserjian RP, Greenberg PL (2024). Molecular taxonomy of myelodysplastic syndromes and its clinical implications[J]. Blood.

[4] Arber DA, Orazi A, Hasserjian R (2016). The 2016 revision to the World Health Organization classification of myeloid neoplasms and acute leukemia[J]. Blood.

[5] Greenberg PL, Tuechler H, Schanz J (2012). Revised international prognostic scoring system for myelodysplastic syndromes[J]. Blood.

[6] Li B, Liu J, Jia Y (2018). Clinical features and biological implications of different U2AF1 mutation types in myelodysplastic syndromes[J]. Genes Chromosomes Cancer.

[7] Zhang Y, Wu J, Qin T (2022). Comparison of the revised 4th (2016) and 5th (2022) editions of the World Health Organization classification of myelodysplastic neoplasms[J]. Leukemia.

[8] 李 冰, 王 静雅, 刘 晋琴 (2017). 靶向测序检测511例骨髓增生异常综合征患者基因突变[J]. 中华血液学杂志.

[9] Awada H, Nagata Y, Goyal A (2019). Invariant phenotype and molecular association of biallelic TET2 mutant myeloid neoplasia[J]. Blood Adv.

[10] Falini B (2023). NPM1-mutated acute myeloid leukemia: New pathogenetic and therapeutic insights and open questions[J]. Am J Hematol.

[11] Zhang Y, Zhang M, Yang L (2007). NPM1 mutations in myelodysplastic syndromes and acute myeloid leukemia with normal karyotype[J]. Leuk Res.

[12] Patel SS, Ho C, Ptashkin RN (2019). Clinicopathologic and genetic characterization of nonacute NPM1-mutated myeloid neoplasms[J]. Blood Adv.

[13] Montalban-Bravo G, Kanagal-Shamanna R, Sasaki K (2019). NPM1 mutations define a specific subgroup of MDS and MDS/MPN patients with favorable outcomes with intensive chemotherapy[J]. Blood Adv.

[14] Gener-Ricos G, Bataller A, Rodriguez-Sevilla JJ (2024). NPM1-mutated myeloid neoplasms are a unique entity not defined by bone marrow blast percentage[J]. Cancer.

[15] 李 璘, 张 悦, 马 晓瑭 (2010). 原发性骨髓增生异常综合征患者NPM1基因突变的研究[J]. 中华血液学杂志.

[16] Menssen AJ, Khanna A, Miller CA (2022). Convergent Clonal Evolution of Signaling Gene Mutations Is a Hallmark of Myelodysplastic Syndrome Progression[J]. Blood Cancer Discov.

[17] Shih L, Huang C, Wang P (2004). Acquisition of FLT3 or N-ras mutations is frequently associated with progression of myelodysplastic syndrome to acute myeloid leukemia[J]. Leukemia.

[18] Takahashi K, Jabbour E, Wang X (2013). Dynamic acquisition of FLT3 or RAS alterations drive a subset of patients with lower risk MDS to secondary AML[J]. Leukemia.

[19] Makishima H, Yoshizato T, Yoshida K (2017). Dynamics of clonal evolution in myelodysplastic syndromes[J]. Nat Genet.

[20] 黄 慧君, 李 冰, 秦 铁军 (2020). 骨髓增生异常综合征RAS基因突变的分子学特征及预后意义[J]. 中华血液学杂志.

[21] 刘 琳琳, 李 冰, 秦 铁军 (2025). 中西方骨髓增生异常肿瘤临床和实验室特征及生存的比较研究[J]. 中华血液学杂志.

[22] Estey E, Hasserjian RP, Döhner H (2022). Distinguishing AML from MDS: a fixed blast percentage may no longer be optimal[J]. Blood.

[23] Bersanelli M, Travaglino E, Meggendorfer M (2021). Classification and Personalized Prognostic Assessment on the Basis of Clinical and Genomic Features in Myelodysplastic Syndromes[J]. J Clin Oncol.

